# Factors affecting the quality of life after total knee arthroplasties: a prospective study

**DOI:** 10.1186/1471-2474-13-116

**Published:** 2012-06-29

**Authors:** Ippolyti Papakostidou, Zoe H Dailiana, Theodoros Papapolychroniou, Lycurgos Liaropoulos, Elias Zintzaras, Theophilos S Karachalios, Konstantinos N Malizos

**Affiliations:** 1 Department of Orthopaedic Surgery, Faculty of Medicine, School of Health Sciences, University of Thessalia, Biopolis 41110, Larissa, Greece; 2 Center for Research and Technology, Thessaly (CERETETH), Department of Biomedical Research & Technology, 41222, Larissa, Greece; 3 Department of Orthopedics, NIMITS Hospital, 10 Monis Petraki Street, Athens, Greece; 4 Center for Health Services Management and Evaluation, Faculty of Nursing, University of Athens, 123 Papadiamantopoulou Street, 11527, Athens, Greece; 5 Department of Biomathematics, Faculty of Medicine, School of Health Sciences, University of Thessalia, Biopolis 41110, Larissa, Greece; 6 The Institute for Clinical Research and Health Policy Studies, Tufts Medical Center, Tufts University School of Medicine, Boston, MA, USA

**Keywords:** Total knee arthroplasty, Quality of life, Osteoarthritis, Rehabilitation

## Abstract

**Background:**

The purpose of the study is to evaluate the self-reported outcomes in the first year after primary total knee arthroplasty (TKA), and to determine factors influencing the quality of life (QoL) 6 weeks, 3, 6, and 12 months after TKA.

**Methods:**

A cohort of patients with knee osteoarthritis undergoing primary TKA at two hospitals (a regional university hospital and a capital’s metropolitan hospital) was prospectively followed for 12 months. Patients were assessed preoperatively and at 4 postoperative time-points, with the use of self-reported measurements for pain, physical function and depression with the following evaluation tools: Western Ontario and McMaster Osteoarthritis Index [WOMAC], Knee Society Scoring system [KSS], Centre for Epidemiological Studies Depression Scale, [CES-D10] and visual analog scale [VAS] for pain). General linear modelling for repeated measures was used to evaluate the effect of each independent variable including clinical and sociodemographic data. Differences between groups at different time points were tested by the independent samples t-test.

**Results:**

Of the 224 eligible patients, 204 (162 females, mean age 69.2) were included in the analysis. Response rate at one year was 90%. At 6 weeks after surgery, despite improvement in pain and alleviation of the depressive mood, the physical function remained less satisfactory. Females presented lower scores in terms of quality of life, both preoperatively and 6 weeks after TKA. Significant improvement was already experienced at 3 months postoperatively. According to WOMAC, KSS, CES-D10 and pain VAS scores the Qol was significantly improved 12 months after TKA (P < 0.001). CES-D10 score was positively correlated with WOMAC and pain VAS scores at all the time points assessed (P < 0.001). Age, body mass index (BMI), place of residence, level of education and social support were not significant predictors of QoL after TKA.

**Conclusions:**

Patients experienced great improvement in their QoL after TKA in spite of a less satisfactory physical function in the first 6 weeks after surgery, with noticeable differences in the QoL among genders in the same time period. After that period all patients experienced significant improvement for all the measured parameters, until the third postoperative month with smaller changes thereafter.

## Background

Osteoarthritis (OA) is one of the most common causes of chronic pain and functional disability in the elderly and is related to genetic predisposition, environmental factors, lifestyle changes and ageing. The increased life expectancy and the tendency for obesity in younger individuals have lead to an increased prevalence of the symptomatic knee OA with broad variation among different populations[1]. It is reflecting not only genetic differences but also preferences in the physical and lifestyle activities, trauma and the obesity, apart of the methodological variations among the different studies [2-5]. In Greece, the age and sex adjusted, estimated prevalence of symptomatic knee OA is 6.0% (95% CI 5.6–6.4). It is more common in the rural populations (7%) and has higher prevalence in women than men with a ratio of 2.7 to 1 [6]. In patients suffering from OA that does not respond to medical treatment, total knee arthroplasty (TKA) is the most effective surgical procedure to reduce pain, correct the deformity and improve the patient’s quality of life (QoL) [7-11].

Numerous follow-up studies after TKA reported that several socio-demographic and clinical variables, such as pre-intervention QoL scores, age, gender, obesity, social support, the number of comorbidities and the status of the mental health, may influence the outcome [10-14].

This study prospectively evaluates the QoL after TKA, in a cohort of Greek patients. The objectives of the present study are to investigate the effect of patients’ demographic and clinical characteristics on the three dimensions of QoL (bodily pain, physical function and mental health) preoperatively and in a 12-month post-TKA period, and to identify disparities in the clinical outcome based on location of residence, educational status and social support.

## Methods

The cohort consisted of patients from the orthopaedic departments of two hospitals: the University Hospital of Larissa, located in central Greece, and the Veteran’s Hospital, located in downtown Athens. The duration of the follow-up was 12 months. The patient population of the University Hospital covered a broad spectrum of origin from rural to urban areas of the region while the population of the Veteran’s Hospital of Athens originated from urban near by areas. In Greece, the municipalities in which the largest settlement has less than 2,000 inhabitants are considered rural areas, while semi-urban are considered the areas with 2,000-10,000 inhabitants, and urban the areas with population larger than 10,000 [6,14]. Patients were included in the study if they fulfilled the following criteria: they suffered from severe knee arthritis (OA or traumatic) and were scheduled to undergo primary TKA, were speaking the Greek language and had an adequate hearing and cognitive function. Patients with knee replacement due to inflammatory diseases, severe neurological, cardiac, and psychiatric comorbidities that would significantly compromise physical function and those residing in long-term institutions and nursery houses were excluded. All those who agreed to participate in the study gave a written consent and agreed upon the follow-up evaluations at pre-scheduled intervals, during the first postoperative year. The study was approved from the hospitals ethics committees.

During the recruitment period 252 patients underwent primary TKA, but 27 did not meet the criteria, 5 refused to participate, and 16 underwent contralateral TKA during the follow-up period and were excluded from the study. The remaining 204 patients, 162 women and 42 men, were included and further evaluated. All patients started walking on crutches or a walker the second postoperative day and they were able to walk independently and in good balance before discharge from the hospital.

### Study design and data collection

The study design was prospective, with baseline measurements at the day before surgery and postoperative follow-up with personal contacts at 6 weeks, 3, 6, and 12 months. The information on sociodemographic characteristics of the patients such as age, gender, educational level, place of residence and social support status were record on a structured questionnaire. The patients’ social support was determined by their marital and living status. Patients who stated in the pre-intervention questionnaire that there were married or were living with someone, were defined as having more social support than those who were single and living alone.

The clinical parameters included specific diagnosis, body mass index (BMI), previous major joint arthroplasty on the contralateral knee and Charlson Comorbidity Index score [15]. Data on perioperative and postoperative complications, waiting-time to surgery in weeks and length of hospital stay in days, were also included. Information regarding hospital readmissions, post-hospital care and destination at discharge, as well as rehabilitation within 2 months of surgery, were gathered at the follow-up interviews.

One investigator (IP), who was not involved in the direct care of the participants, administered the questionnaires in face-to-face interviews and evaluated the different parameters (e.g. range of motion). For patients who were unable to read Greek because of illiteracy the questions were read-out by the interviewer.

### Quality of life measurements

Four validated measurement tools were used: the Western Ontario and McMaster’s Universities (WOMAC) Osteoarthritis Index, the Knee Society Score (KSS), the Centre for Epidemiological Studies Depression Scale, short form (CES-D10), and the visual analogue scale (VAS) for pain.

The WOMAC is a well-known, disease-specific instrument for measuring clinical outcome in patients treated for knee osteoarthritis [16,17]. Using a Likert scale, patients rate themselves on multiple items grouped in three domains: pain, stiffness and difficulty in function. The maximum score is 20 points for pain, 8 for stiffness and 68 points for clinical function. Higher scores indicate greater difficulty.

The KSS consists of two scores, a knee score and a functioning score, both ranging from 0 (worst health or functioning) to 100 (best health or functioning) [18]. The knee score reflects an objective measurement as well as patient-reported pain severity. The function score reflects patient-reported walking distance and stair-climbing and makes deductions for use of a walking aid, with 100 representing unlimited walking distance and normal stair-climbing without use of an aid.

The CES-D short form is a 10-item self-reported measure of depressive symptoms commonly observed in older adults with chronic pain and was employed for independent assessments of depression and pain at each evaluation in order to measure potential improvement of mood after the surgical intervention. Scores range from 0 to 30, with higher scores indicating a higher frequency of current depressive symptoms experienced during the past week. Investigators have used a validated cut off score of 10 to differentiate clinically depressed from non-depressed patients. [19-21].

The VAS is a commonly used assessment tool measurement for pain [22,23]. Individuals are asked to mark on a 10-cm line their pain rating, with 0 representing no pain and 10 representing extreme pain. The WOMAC, CES-D10 and VAS were used at baseline and at all follow-up contacts whereas the KSS was used at baseline and 3, 6, and 12 months.

### Statistical analysis

The independent variables were age, gender, BMI (under and over 30 kg/m^2^), level of education (elementary/ less and high), place of residence (rural and urban/semi-urban), social support (married /living with someone and otherwise), preoperative WOMAC, KSS, CES-D10, and VAS scores. The data were summarised as means ± S.D. or percentages. The effect of each independent variable was analyzed separately for the WOMAC and KSS questionnaires, and the CES-D10 and VAS scores, in time (baseline, 6 weeks, 3, 6, 12 months) using general linear modelling for repeated measures and post-hoc tests with Bonferroni’s correction. A general linear model multivariable analysis was used to estimate the effects of all factors of interest (gender age, BMI, level of education, social support and place of residence) on each response variable (QoL questionnaires) at 12 months postoperatively. The corresponding preoperative scores were used as covariates. The independent-sample t-test was performed to compare two groups' scores on the same variable. Pearson’s correlation coefficients were calculated to examine the relation between changes in WOMAC pain and VAS pain scores with changes in CES-D10 score. Effect sizes (ES) were calculated for the different outcome measures using the formula ES = mean change/SD of preoperative scores. The effect size is a standardised measure that provides information regarding the magnitude of change before and after TKA. An effect size of 0.8 or greater is considered large [24].

A value of P < 0.05 was considered statistically significant. Statistical analyses were performed using SPSS 13.0.

## Results

The response rates at 6 weeks, 3 months, 6 months and 12 months follow-up were at 98.5%, 97%, 94.6%, and 90.2% respectively.

### Patients’ characteristics

Table 1 summarizes the main characteristics of the study group population. The majority of patients underwent the procedure under spinal or epidural anaesthesia (99%). The surgical procedure lasted from 75 to 180 minutes (91.9 ± 19.9). The length of stay (LOS) in hospital postoperatively varied between 6 and 16 days (6.68 ± 1.3). Patients received thromboembolic prophylaxis, with low molecular weight heparin (LMWH) for 30 days postoperatively. The most frequently cited in-hospital complications were urinary tract infection (n = 3). Two readmissions during the first two preoperative months were directly related to the prosthesis and required manipulation of the knee under general anesthesia for inadequate range of joint motion.

**Table 1:** Characteristics of participants (n = 204)

**Demographics**	
Age, years †	69.17 ± 6.69
Female, No. (%)	162 (79.4)
Absence of social support, No. (%)	36 (17.6)
Education: Elementary or less, No. (%)	130 (63.7)
Residence: Rural, No. (%)	86 (42.1)
**Medical status**	
Osteoarthritis, No. (%)	196 (96)
Prior contralateral TKA, No. (%)	44 (21.5)
Charlson comorbidity scale ▫	1.6 (1.5)
Body mass index ≥30, No. (%) ††	108 (52.9)
Waiting time (weeks) †	13.6 ± 10.9 median 12
**Complications (in- hospital),** No. (%)	
Cerebral hemorrhage	1 (0.5)
Pulmonary embolism	1 (0.5)
Deep venous thrombosis	1 (0.5)
Urinary tract infection	3 (1.5)
Wound infection	1 (0.5)
Delirium	1 (0.5)
Mortality within 60 post-op days	2 (1)

† Values are mean ± SD; ▫ Charlson comorbidity scale, 0–27 (higher scores indicate more comorbid illness).

††Weight in kilograms divided by height in meters squared.

### Rehabilitation

The majority of the patients (176) went home after discharge and followed a rehabilitation program supported by a physiotherapist with 12 sessions of physical therapy in 6 weeks period, starting the day after discharge. The remaining 28 patients were transferred to rehabilitation centers with a mean length of stay 19.3 ± (3.3) days and received a similar program of physiotherapy.

### Correlations with preoperative QoL

The preoperative scores of the studied domains are analyzed in Table 2. Analysis of the WOMAC domains showed that women reported worse preoperative bodily pain (12.2 ± 3.8 versus 10.3 ± 4.5 units; P = 0.007), physical function (39.6 ± 10.1 versus 34.7 ± 14.1 units; P = 0.01), and stiffness (4.2 ± 1.5 versus 3.6 ± 1.8 units; P = 0.02) as compared with men. Patients with BMI ≥ 30 kg/m^2^ had worse preoperative bodily pain (12.4 ± 3.8 versus 11.1 ± 4.1 units; P = 0.04) and poorer preoperative physical function (40.5 ±10.6 versus 36.2 ± 11.6 units; P = 0.01) than did the patients with BMI < 30 kg/m^2^. There were no statistically significant differences in WOMAC domains across age, educational status, and residence or social support categories.

**Table 2:** Preoperative WOMAC, KSS, CES-D10, and pain VAS scores according to patients’ characteristics*

	**Gender**		**Age**		**BMI**		**Level of education**		**Social support**		**Residence**	
	**male**	**female**	**<65 years**	**≥ 65 years**	**<30**	**≥30**	**low§**	**high**	**yes**	**no**	**rural**	**urban/semi-**
**WOMAC scale**												
Pain	10.3(4.5)	12.2(3.8) ††	12.4(3.4)	11.6(4.2)	11.1(4.1)	12.4(3.8)†	12.3(4.2)	11.7(3.9)	11.8(4.1)	11.7(3.5)	11.7(4.5)	11.9(3.7)
Function	34.7(14.1)	39.6(10.1)†	37.8(12.4)	38.6(10.9)	36.2(11.6)	40.5(10.6)†	42.1(10.7)	37.9(11.3)	38.3(11.9)	39.5(7.3)	38.7(12.7)	38.4(10.3)
Stiffness	3.6(1.8)	4.2(1.5)†	3.9(1.7)	4.1(1.6)	3.9(1.6)	4.2(1.6)	4.6(1.3)	3.9(1.6)	4.1(1.6)	3.9(1.6)	4.1(1.5)	4.0(1.7)
**KSS**												
Knee score	45.3(18.8)	39.2(17.6)†	38.8(19.3)	41.4(17.6)	41.3(19.6)	39.9(16.6)	38.9(15.8)	41.6(18.2)	40.41(18.4)	41.1(16.0)	40.9(18.3)	40.3(17.9)
Function score	37.5(13.2)	32.5(11.7)†	37.5(15.9)	33.9(10.4)	36.1(13.7)	33.03(10.4)	33.6(10.5)	34.61(12.4)	34.8 (11.9)	32.5(12.7)	34.1(11.2)	34.7(12.7)
**CES-D10**	5.9(6.1)	10.4(6.4)††	10.0(6.5)	8.8(6.6)	8.1(0.7)	9.9(0.6)	9.8(6.8)	8.8(6.7)	8.7(6.6)	11.3(6.1)†	9.5(6.8)	8.9(6.5)
**Pain VAS**	7.9(2.1)	9.0(1.6)††	9.0(1.4)	8.6(1.9)	8.5(2.0)	9.0(1.4)	9.9(1.6)	8.7(1.8)	8.6(1.8)	9.2(1.3)	8.6(2.0)	8.8(1.6)

* Values are mean ± SD; † P < 0.05, †† P < 0.01; §Elementary or less.

WOMAC scores: pain 0–20, function 0–68, stiffness 0–8; higher scores indicate greater difficulty.

KSS: Knee score 100–0, Functional score 100–0, higher scores indicate better health state.

CES-D10 scores: > 10 indicate depression; Pain VAS: 0–10, better to worse.

Analysis of the KSS questionnaire showed that women had significantly worse scores as compared with men (Knee score 39.2 ± 17.6 versus 45.3 ± 18.8 units; P = 0.02 and Functional score 32.5 ± 11.7 versus 37.5 ± 13.2 units; P = 0.04). No significant differences were detected in KSS domains across, age, BMI, educational status, place of residence or social support categories.

According to CES-D10, depression (score of 10) was detected preoperatively in 44.5% of patients (9 males and 81 females). Women and those without social support had more depressive symptoms compared to male patients and to those having social support; these differences were statistically significant (P = 0.003, P = 0.04 respectively). Finally, VAS pain detected significantly higher scores in women than their counterparts (9.0 ± 1.6 versus 7.9 ± 2.1 units; P = 0.004).

### Postoperative changes in QoL over time

Table 3 gives the mean change in scores with 95% confidence intervals obtained for each measurement at the five time points. Table 3 gives (preoperative and at 6 weeks, 3, 6, and 12 months postoperatively). All the scores showed a significant improvement at the final follow-up (P<0.001). Table 4 analyses the 12-month post-op scores according to the examined independent variables.

**Table 3:** Differences in QoL scores between the examine intervals (baseline,6 weeks,3 -6-12-months)*

	**Baseline-6 weeks**	**6 weeks-3 months**	**3 months-6 months**	**6 months-12 months**	**Baseline-12 months**	**Effect size Baseline-12 m**
**WOMAC**						
Pain	3.1(1.9-4.2)†	4.0(2.9-5.3)†	1.8(1.4-2.4)†	1.3(0.5-1.0)†	10.2(9.4-11.5)††	2.5
Function	1.8(−1.2- 5.4)	16.5(13.5-18.8)†	9.4(7.4(11.2)†	3.5(5.0-1.9)†	31.2(28.8-34.7)††	2.7
Stiffness	0.6(−0.1-1.4)	1.0(0.7-1.3)†	0.8 (0.5-0.9)†	0.9 (0.6-1.0)†	3.3(2.8-3.7)††	2.0
**KSS**						
Knee score	-	−36.7(−41.2- -32.3)†	−8.6(−11.1- -6.1)†	−2.9(1.1-4.7)†	−48.7(−54.5-45.2)††	2.7
Function score	-	−17.2 (−20.4- -3.7)†	−11.5(−14.2 -8.8)†	−5.6(−7.7 -3.4)†	−34.2(−37.5-30.8)††	2.8
**Pain VAS**	2.6(2.0-3.1)†	2.0(1.6-2.5)†	1.7(1.2-2.1)†	0.8(−0.4 - 1.1)	7.1(6.5-7.6)††	3.9
**CES-D10**	1.5(−0.3-3.1)†	2.8(1.6-4.1)†	1.2(0.3 -2.3)†	1.0(−0.3-1.8)†	6.6(4.9-7.9)††	1.0

*Values are mean (95% confidence interval of the difference); Repeated measures GLM with post-hoc Bonferroni’s correction.

† P < 0.05 †† P < 0.001; - Not applicable.

WOMAC scores:pain 0–20,function 0–68,stiffness 0–8,higher scores indicate greater difficulty.

KSS: Knee score 100–0, Functional score 100–0, higher scores indicate a better health state.

CES-D10 scores: > 10 indicate depression; Pain VAS: 0–10, better to worse.

**Table 4:** 12 months postoperative scores according to patients’ characteristics*

	**Gender**		**Age**		**BMI**		**Level of education**		**Social support**		**Residence**	
	**male**	**female**	**<65 years**	**≥ 65 years**	**<30**	**≥30**	**Low§**	**high**	**yes**	**no**	**rural**	**urban/semi**
**WOMAC scale**												
Pain	1.6(2.4)	1.4(2.4)	1.3(1.9)	1.7(2.5 )	1.5(2.2)	1.7(2.6)	1.1(1.5)	1.7(2.5)	1.5(2.3)	2.1(2.9)	1.7(2.4)	2.1(2.8)
Function	7.5(9.4)	7.2(8.1)	5.3(6.7)	7.9(8.8)	6.4(8.1)	7.9(8.6)	6.4(7.7)	7.4(8.5)	7.0(8.5)	8.4(8.1)	7.2(8.1)	8.3(9.3)
Stiffness	0.7(0.9)	0.8(1.1)	0.7(0.9)	0.9(1.1)	0.8(1.0)	0.9(1.0)	0.9(1.1)	0.5(0.7)	0.8(1.0)	0.9(1.1)	0.9(1.1)	0.7(.09)
**KSS**												
Knee score	89.2(12.1)	89.3(10.6)	90.9(10.3)	88.64(11.1)	89.8(10.5)	88.7(10.9)	92.3(5.7)	88.7(11.5)	90.6(9.5)	89.4(10.6)	87.0(12.6)	90.6(9.5)
Function score	69.3(15.0)	68.4(13.9)	72.1(13.2)	67.4(14.1)	69.6(14.1)	67.7(14.1)	67.1(12.7)	68.8(14.4)	69.0(13.9)	66.5(14.8)	65.2(13.3)	70.8(14.2)
**CES-D10**	1.7(4.6)	3.0(4.5)†	2.1(3.8)	2.9(4.7)	2.3(4.1)	2.9(4.8)	1.9(3.4)	2.8(4.7)	2.6(4.4)	3.3(5.2)	3.1(5.0)	2.4(4.5)
**Pain VAS**	1.5(2.1)	1.7(2.1)	1.(2.0)	1.7(2.1)	1.5(2.1)	1.7(2.0)	1.3(1.8)	1.7(2.1)	1.5(2.0)	2.0(2.4)	1.9(2.2)	1.4(2.0)

*Values are mean ± SD; † P < 0.05; §Elementary or less.

WOMAC scores: pain 0–20, function 0–68, stiffness 0–8; higher scores indicate greater difficulty.

KSS: Knee score 100–0, Functional score 100–0, higher scores indicate better health state.

CES-D10 scores: > 10 indicate depression; Pain VAS: 0–10, better to worse.

#### WOMAC scores

All groups of patients showed a statistically significant improvement in WOMAC domains between the pre- and the 12-month post-operative assessments. Patients improved from a bodily pain score of 11.8 ± 4.0 units preoperatively to a score of 1.6 ± 2.4 units (P < 0.001) 12 months postoperatively. Similar gains were observed for WOMAC function score (38.5 ± 11.3 to 7.3 ± 8.4 units; P < 0.001), and for stiffness score (4.5 ± 1.6 to 1.2 ± 1.0 units; P <0.001). All WOMAC domains showed improvement at each follow-up visit (6 weeks, 3, 6, and 12 months).

**Figure 1:**
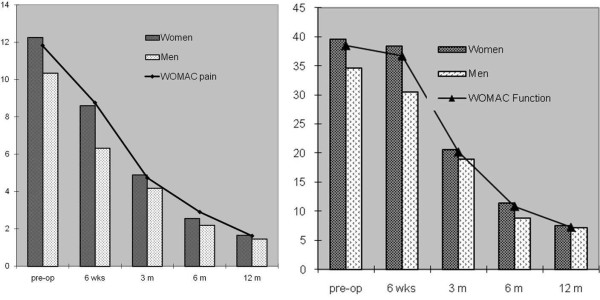
WOMAC pain and function scores according to gender.

Table 3 also demonstrates that at 6 weeks postoperative follow-up patients did not improve their physical function (P = 0.6) and stiffness scores (P = 0.1). There were no significant differences in WOMAC domains across age, BMI, education, residence or social support categories, except the significantly worse scores that women exhibited in the domains of pain (8.6 ± 3.7 versus 6.0 ± 2.8 units; P = 0.007) and physical function ( 38.4 ± 11.8 versus 30.5 ± 12.5 units; P = 0.007) when compared with men, 6 weeks postoperatively (Figure 1). After that women and men exhibited similar gains (Table 4).

Regarding the three WOMAC domains, the multivariable analysis showed that the only predictive variables of better outcome were the baseline scores for pain and function (Table 5).

**Table 5:** Multivariable analysis of post-operative (12 months) changes in WOMAC domains

WOMAC *						
	**Pain**		**Function**		**Stiffness**	
**Variables**	**Diff† (95%CI)**	**P-value**	**Diff (95%CI)**	**P-value**	**Diff (95%CI)**	**P-value**
**Gender** (Male vs Female)	0.02 (-0.84, 0.88)	0.2	1.30 (-1.68, 4.28)	0`.4	-0.15 (-0.52, 0.23)	0.4
**Age** (< 65 vs ≥65)	-0.48 (-1.28, 0.31)	0.9	-2.47 (-5.23, 0.28)	0.07	-0.18 (-0.53, 0.17)	0.3
**BMI** <30 vs ≥30	-0.15 (-0.85, 0.54)	0.6	-0.97 (-3.41, 1.46)	0.4	-0.06 (-0.37, 0.24)	0.4
**Education** (Low§ vs High)	-0.72 (-1.69, 0.44)	0.06	-3.08 (-6.76, 0.59)	0.09	-0.16 (-0.70, 0.28)	0.8
**Social Support** (Yes vs No)	-0.86 (-1.82, 0.09)	0.07	-2.24 (-5.55, 1.06)	0.1	-0.17 (-0.59, 0.25)	0.4
**Residence** (Rural vs Urban/semi)	-0.52 (-1.10, 0.42)	0.05	-2.25 (-4.87, 0.38)	0.09	-0.15 (-0.59, 0.22)	0.06
Pre-intervention Pain	0.10 (2.29, 0.02)	**0.02**	-	-	-	-
Pre-intervention Function	-	-	0.17 (0.06, 0.28)	**0.002**	-	-
Pre-intervention Stiffness	-	-	-	-	0.07 (-0.02, 0.17)	0.1

*WOMAC scores: pain 0–20, function 0–68, stiffness 0–8; higher scores indicate greater difficulty.

† Estimated differences between categories after adjustment by all other variables.

§ Elementary or less; - Not applicable.

#### KSS scores

The improvement compared with baseline was statistically significant at 3, 6, and 12 months. In Knee score, patients were improved from 40.9 ± 18.0 units preoperatively to 89.3 ± 10.9 units 12 months postoperatively (P < 0.001). Similar pattern was observed for Function score: it improved from 34.4 ±12.2 preoperatively to 68.6 ± 14.1 units (P <0.001) 12 months postoperatively. In both KSS domains, there is no significant effect for gender, age, BMI, social support, education and place of residence at any postoperative follow-up interval (Table 4). The multivariable analysis has not disclosed predictive variables of KSS outcome,(data not shown).

#### Pain VAS and CES-D10 scores

Pain VAS score significantly improved from a preoperative score of 8.7 ± 1.8 units to 12-month postoperative score of 1.6 ± 2.1 units (P < 0.001). There were no statistically significant differences across age, BMI, education, residence or social support categories (Table 4).

Similar gains were observed for CES-D10 scores. Patients’ mean ± SD score improved from 9.1 ± 6.6 preoperatively to 2.5 ± 4.5 units (P < 0.001) at 12 months after surgery.

High scores, indicative depression were not found among the different groups of the study, during the postoperative follow-up. However, women had significantly worse scores when compared with men over time (P < 0.05; Table 4). Strong positive correlations were found when the correlation coefficient of CES-D10, WOMAC and VAS pain scores were examined preoperatively and at the 4 follow-up periods (P < 0.001; Table 6). Regarding the multivariable analysis, only the baseline CES-D10 score was predictive of better outcome (P = 0.02; data not shown).

**Table 6:** Pearson’s Correlation Coefficient between CES-D10 and WOMAC pain score and pain VAS

	**CES-D10 vs WOMAC pain**	**CES-D10 vs VAS pain**
Preoperative	0.463**	0.371**
6 weeks post-op	0.630**	0.480**
3 months post-op	0.645**	0.627**
6 months post-op	0.629**	0.568**
12 months post-op	0.738**	0.656**

## Discussion

The present study evaluated prospectively the QoL after TKA in a cohort of 204 patients, and examined the effect of socio-demographic characteristics.

Our study demonstrated that the quality of life in patients with end stage arthritic knees presents significant differences among genders [25-27]. WOMAC scores were significantly worse in women preoperatively and at 6 weeks after surgery. By the third postoperative month the WOMAC score differences among genders fully resolved [28,29]. Also according to KSS measuring scale, women had significantly lower preoperative scores, but with equivalent improvement postoperatively when compared with men, at any follow-up interval. These gender differences observed preoperatively have been attributed to a delayed access of the women to surgical management for their arthritic knee until their symptoms were more severe than in men [7,29-31]. The lower rate of the improvement observed at the 6 weeks follow-up in women may be due to the more severe preoperative disability of women and thus, to the longer periods needed to achieve improvement similar to men.

This study also demonstrated that older age does not affect negatively the functional outcome after TKA [7,10,13,25,32]. However, the small number of older patients precludes any definite conclusions. According to the literature, the effect of obesity on the outcome of knee replacement is unclear [33-36]. In the present study obese patients reported more pain, functional limitations and depressed mood before the surgical procedure, but obesity was not a significant predictor of pain and functional limitations one year after the index operation, suggesting that obesity is not related to the short-term outcome. However, the power to detect a significant BMI effect on pain and functional limitations at 12 months postoperatively was limited (power = 12% and 23%, respectively), and therefore, the results should be interpreted with caution. Similarly these findings cannot address long-term concerns regarding potential premature joint failure. It must also be noted that patients with morbid obesity (BMI > 40 kg/m^2^) were not offered the option of TKA from the surgeons of the present series, during the study period.

In the present study, it was hypothesized that patients living in rural areas, which reflects a lower socioeconomic and educational status and limited or no access to rehabilitation facilities, were more likely to have access barriers or underutilization of health care services especially in the early postoperative period that would consequently impact their outcome after a TKA [37-39]. According to our findings, residents of rural areas do not appear to have a worse outcome following TKA. A possible explanation is that access to all public medical services is equivalent and is not limited for patients of lower socioeconomic background. In addition, routine visits to the outpatient department during the follow-up period also offered the opportunity of a close patient-surgeon contact, provided information about the rehabilitation process even in cases with own care for physical therapy, and eliminated patients insecurity and lack of knowledge and care.

Some studies demonstrated that social support might play an important role in moderating the effects of pain, physical disability, and depression in patients with osteoarthritis [40,41]. In addition, patients consider social support as an important factor when they are deciding the operative treatment with TKA and its timing [42]. In the present study, patients that were married or living with others did not have a better QoL compared to those not married and living alone [43]. A possible explanation is that other family members, friends or neighbours take care of those patients with weak social support, as the Greek public health system does not offer formal community services as district nurses, home help and care or day centre attendance. The mechanism of social support on TKA outcomes needs further investigation.

Chronic pain and depression are closely related to each other and many studies attempted to reveal the causality [44-47]. Based on the assessment tools employed in our study we found a high prevalence of depression among patients preoperatively (44.2%; 9 males and 81 females) [48]. However after the surgical procedure, levels of depression changed significantly over the course of the study, and 12 months after surgery a small amount of patients (7.35%; 3males and 12 females) remained in depressed mood. This suggests that depressed mood might be related to the levels of chronic pain and disability and is amenable to significant improvement when pain is alleviated and function is resumed after successful TKA. More evidence is needed to draw safe conclusions regarding this association.

At 12 months postoperatively the TKA lead to a significant reduction in pain, stiffness, functional disability and depressed mood with the pain dimension showing the greatest improvement, although only 5% of patients complained for mild chronic pain without obvious concomitant clinical or radiographic sights that might explain this symptom. The greatest improvement was seen in all measurements within the first 3 postoperative months with smaller changes thereafter. However, at 6 weeks after surgery patients still experienced functional limitations and stiffness despite significant improvements in pain. The finding of limited early functional recovery is consistent with the findings from other studies suggesting that after an initial period of functional limitation patients improved by 3 months after surgery [10,33]. These findings have important implications for patients and their families regarding the expected physical dependencies after surgery and should stimulate a physician – patient discussion about the particular needs for assistance in the daily routine mainly for single individuals for the first 2 months after discharge from the hospital.

Based on our data, we can conclude that the baseline WOMAC pain and function scores are a strong determinant of the respective post-operative scores at 12 months. The same conclusions apply to the CES-D10 score.

We acknowledge however that this study was presents certain limitations such as the involvement of only two centres; therefore a multicenter research is needed for generalization of the results. In addition, the low proportion of males and the narrow age range of our patients, limited the usefulness of the results with respect to gender and age. The social support variable was created based upon the patients-reported preoperative living and marital status, which is only a crude measure of social support. In addition, the patients’ postoperative living conditions and marital status was not specifically investigated. Further studies need to explore these variables. The strengths of the study are its prospective design, the high rate of return to follow-up (90.2% at one year) and the use of a trained independent research assistant who recruited patients and followed them at each assessment.

## Conclusions

Our results demonstrated that quality of life in 95% of the patients with severe knee osteoarthritis was significantly improved in the first three months after uncomplicated TKA and thereafter. Women presented with worst pain, physical function and depressed mood preoperatively and they should be informed for that benefit when considering TKA earlier in the course of their arthritis. However 5% of patients stated that they experienced some mild symptoms 12 months after surgery. Age, BMI, level of education, social support and place of residence do not seem to influence knee replacement outcomes. Despite significantly milder pain and better physical function in the first 6 weeks postoperatively, for both men and women, the patients may need another person’s assistance for this time period after surgery. Finally, the finding that depressed mood has a strong positive correlation with chronic and more severe pain and functional limitation warrants further examination.

## Abbreviations

TKA, Total knee arthroplasty; QoL, Quality of life; WOMAC, Western Ontario and McMaster Osteoarthritis Index; KSS, Knee Society Scoring system; CES-D10, Centre for Epidemiological Studies Depression Scale, short form; VAS, Visual analog scale; BMI, Body mass index; OA, Osteoarthritis.

## Competing interests

The authors declare that they have no competing interests.

## Authors' contributions

IP participated in the concept and design of the study, and the acquisition and the interpretation of the data and wrote the manuscript. ZHD participated in the concept and design of the study, reviewed the findings, and revised the manuscript. TP participated in the design of the study and helped draft the manuscript. LL participated in the concept and design of the study and helped draft the manuscript. EZ participated in the concept and design of the study, performed the statistical analysis and revised the manuscript. TSK participated in the design of the study and helped draft the manuscript. KNM participated in the concept and design of the study, critically reviewed the findings and revised the manuscripts’ final version. All authors read and approved the final manuscript.

## Pre-publication history

The pre-publication history for this paper can be accessed here:

http://www.biomedcentral.com/1471-2474/13/116/prepub
